# High-stress surgery in severely frail patients: a case report of personalized and multidisciplinary shared decision-making

**DOI:** 10.1186/s44158-023-00113-7

**Published:** 2023-08-14

**Authors:** Andrea Sanna, Sara Miori, Sergio Lassola, Michele Umbrello, Silvia De Rosa, Giacomo Bellani

**Affiliations:** 1 Department of Anesthesia and Intensive Care, Santa Chiara Hospital, Trento, Italy; 2 Department of Intensive Care Unit at San Carlo, Borromeo University Hospital, Milan, Italy; 3 Centre for Medical Sciences — CISMed, University of Trento, Trento, Italy

**Keywords:** Frailty, Disability, Shared decision-making, Case report

## Abstract

We report the case of a 65-year-old severely disabled man diagnosed with advanced renal neoplasm who was scheduled for major urologic surgery. Through a multidisciplinary approach, a shared decision-making process and mutual listening of all the health professionals involved, it was decided not to have major surgery due to the high risk of worsening the patient’s health and quality of life.

## Background

Patients with a severe psychophysical disability who need surgery represent a significant challenge in perioperative assessment that requires a personalized interdisciplinary approach. DSM V defines *intellectual disability* as an impairment of general mental abilities, impacting adaptive functioning in three domains: conceptual, social and practical [1]; it is an existential human experience that relates to a context of relational, physical and mental complexity. The onset of a severe disease exposes the subject to a further risk of worsening her/his frailty due to the disease itself and the planned therapeutic approach [2]. Major surgery may lead to a significant worsening in the quality of life due to the complexity of the postoperative course [3]. The anaesthesiologist is a crucial figure in related-surgery risk stratification, and a multidisciplinary approach involving surgeons and other healthcare professionals are essential in tailoring the most suitable and valuable diagnostic-therapeutic approach to guarantee a benefit in terms of health and quality of life.

## Case description

We report the case of a 65-year-old man suffering from severe mental retardation, autism spectrum disorder, blindness, hearing loss and epilepsy. He had been living in a nursing home for severely disabled people since he was 15. The man was in a protected apartment together with four adults suffering from serious physical and mental disabilities, assisted day and night by social and health workers. At the time of the anaesthesiologic evaluation, he was not able to walk independently and lived between bed and armchair, with a need for assistance for all daily activities; he was affected by severe dysphagia, unable to feed himself and suffering from urinary and faecal incontinence independently. His disability severely limited his social and relationship life, as he could not express any understandable verbal language and minimal human contact (limited to healthcare workers). In recent months, he had been intolerant to any medical procedure (including blood drawing or intravenous infusions).

The patient had no family support or reference to rely upon, and he did not receive any courtesy visit, being legally represented by a lawyer, appointed by the local court. Hence, it was impossible to identify a stable and reliable caregiver, and no advance directives for treatment were known or legally recorded. The most clinically informed and emotionally connected person was the head nurse of the home, who, thanks to the continuity of care guaranteed for decades, represented the reference person for daily assistance, empathy, and the historical memory of the patient’s life.

Over the previous year, the patient suffered a rapid deterioration of his functional abilities and a worsening of his cognitive and relational state until he lost interaction with the nursing home staff or participation in any social activity. In the last period, his reference doctor noticed further deterioration, with decreased body weight, clinical and laboratory signs of malnutrition, anaemia and sacral and trochanteric bedsore. He, therefore, ordered a total body CT scan, which found a voluminous exophytic hypervascularized expansive renal formation, with central necrotic areas and likely infiltration of the psoas muscle; three micronodules at the thoracic level with bilateral pleural effusion were also found.

### Decision path management

**Fig. 1:**
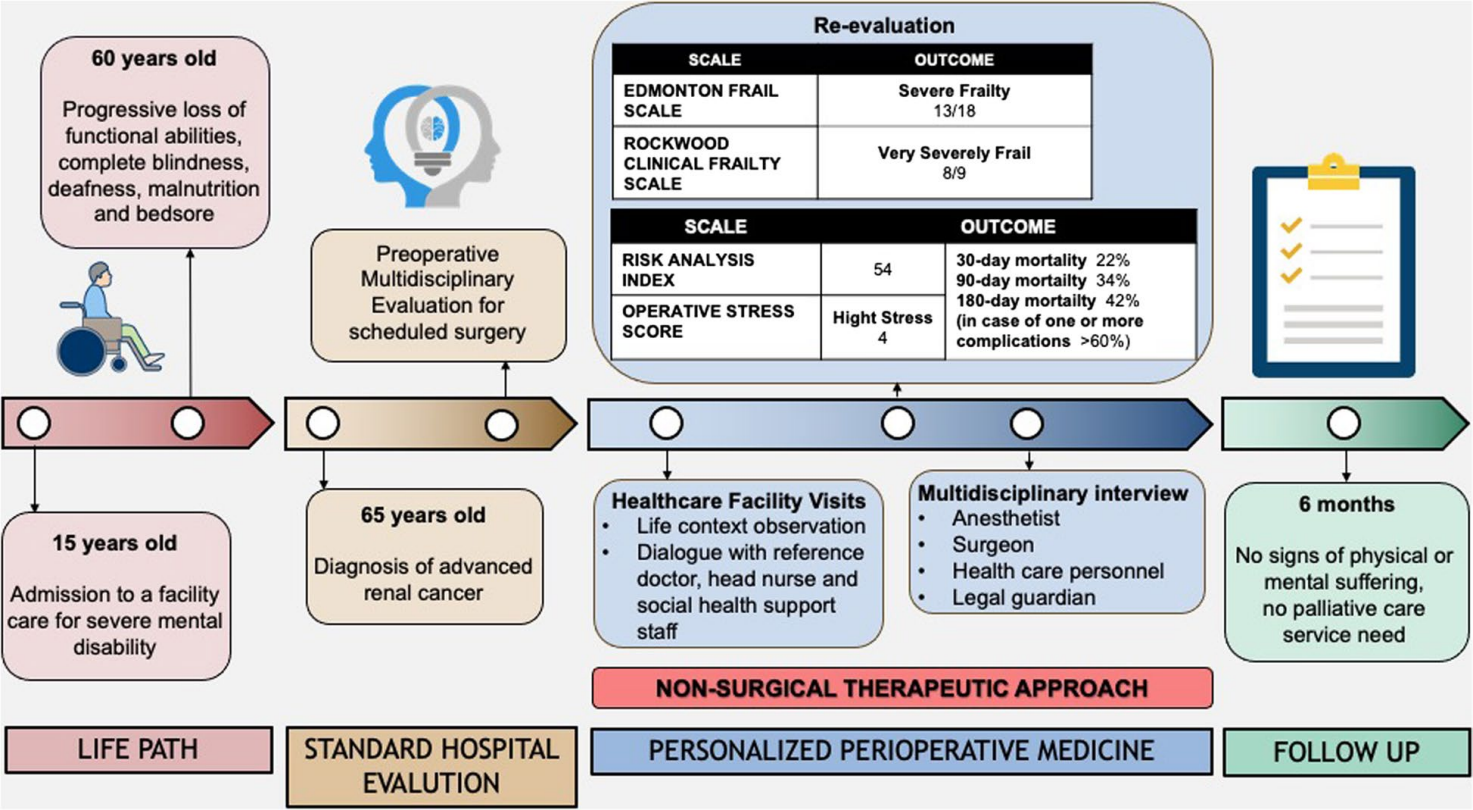
Timeline

After the radiological detection of advanced renal neoplasm, the patient was included in a surgical and anaesthesiologic evaluation process to define the surgical timing. According to the practice of our hospital, the patient was accompanied by an operator of the residence facility to the hospital, where the urologist surgeon and the anesthesiologist evaluated him. Both did not place contraindications to surgery and schedule it; no written informed consent could be signed due to the absence of a legal guardian. A few days later, the absence of written informed consent halted the process. The clinical case was then presented to the anesthesiologist responsible for the perioperative care of patients with severe disabilities (DAMA — Disabled Advanced Medical Assistance — project), who recommended to deepening the clinical evaluation and reevaluating the proposed therapeutic strategy. He contacted the primary physician and the head nurse of the facility (not previously involved), arranging a meeting at the patient’s residence. During the visit, the anaesthesiologist met the operators taking care of the patient’s daily life and observed the patient’s life context. Subsequently, a joint meeting was organized between the anaesthesiologist, urologist, doctor, and head nurse of the facility to deepen the perspectives of care, the expectations in terms of improvement of health, the present and future living conditions and the possible therapeutic alternatives, getting to the conclusion that surgery would not have guaranteed to the patient any benefits in terms of health and quality of life. The lawyer acting as the patient’s legal guardian was also involved, and he did not express any opposition (Fig. 1).

## Discussion

According to the World Health Organization, one in six people worldwide experience significant disability (16% of the global population) [4]. These people are exposed to healthcare inequalities, stigma and discrimination, as recently confirmed during the COVID-19 pandemic [5–7]. Frailty is a state of vulnerability to poor resolution of homeostasis following stress and is a consequence of the cumulative decline in multiple physiological systems over a lifespan [8]; it identifies those patients who, regardless of age, are at greater risk of dying in the subsequent 5 years due to a decline in physiological reserves, in terms of physical, cognitive, social and psychological functions [9]. Frail and very frail patients [3] and those who are affected by intellectual disability [2] are more exposed to postoperative major complications, mortality, morbidity, length of stay and use of health resources [10]. Surgical outcomes are significantly influenced by patients’ overall health, function and life expectancy; at a preoperative visit, it is essential to establish the patient’s goals and preferences and to determine whether the risks and benefits of surgery match these goals and preferences, and a comprehensive approach in frail older patients should also assess patients ‘social support system [11], because these are a critical component of discharge planning [12]. Unfortunately, if the patient's decision-making abilities are impaired, it is impossible to address his preferences on priorities of life (living as long as possible over maintaining independence) or discuss alternatives to surgery.

Our case report shows that even without absolute contraindications to a possible surgical path, it is essential to consider the extreme frailty given by the starting clinical assistance conditions and the surgical stress to which the patient will be exposed. In the multidisciplinary evaluation of a frail patient, it is essential to use objective, reproducible and comparable scores; we evaluated the frailty grade of our patient with two scales: Rockwood Clinical Frailty Scale [13] that assigned 8 points on 9 and the Edmonton Frail Scale [14], with 13 points on 18; both led us to consider our patient extremely frail and thus exposed to a high risk of major post-operative complications. However, to complete the assessment, it is essential to consider type of surgery, type and risk of anaesthesia, recovery time and alternatives to surgery (including palliative care) [11] and discuss if patients do not want resuscitation in the event of significant postoperative complications (if feasible) [15].

Indeed, as demonstrated by Shinall Jr. et al. [3], if we consider our patient’s baseline condition (Risk Analysis Index score 54) and the level of surgical stress related to nephrectomy (Operative Stress Score 4), predictive models estimate mortality at 30, 90 and 180 days of 22%, 34% and 42%, respectively. In case of one or more complications in the postoperative phase, mortality would increase significantly, reaching percentages of over 60% at 180 days. Notwithstanding the limitations of predictive models, these data suggest that in the surgical path, the medical staff involved must consider frailty to determine whether a surgical procedure is appropriate and what can be done during the perioperative management.

The decision-making process with patients with severe mental disabilities is highly challenging due to the lack of best practices or guidelines [16]; even if the United Nations (UN) Convention on the Rights of Persons with Disabilities (CRPD) stresses the importance of respect for the “will and preferences” of the person with a disability, often the clinical conditions of the patient or the social context do not allow to recognize them [17]. Shared-decision making is thus the only helpful care paradigm that facilitates treatment agreement by building consensus and sharing information [18], reducing health disparities and moving towards personalized medicine. However, it requires culture change and reconfiguration of services.

The multidisciplinary approach and mutual listening replaced the lack of communication with the patient, who, due to the severe disability, could not express his own opinion. A further difficulty was the absence of a family member who coincided with legal protection or represented patient’s will. However, the involvement of the health professionals who care for the patient daily and the direct vision of the life context added critical elements to the final decision. Dialogue, sharing opinions and visions of the disease and the patient's life path allowed us to take a step towards the culture of professionalism [19].

The limits of this case are related to the initial management misunderstanding and lack of communication between anaesthesiologists, surgeons and professionals of the nursing home and the absence of a formal opinion of the ethics committee. Moreover, we assessed frailty with Rockwood Clinical Frailty Scale and the Edmonton Frail Scale, the patient’s baseline condition with Risk Analysis Index and surgical stress level with Operative Stress Score, but we could have also used one of the several prognostic models available at http://www.ePrognosis.org to estimate patients’ prognosis and life expectancy or quantify the risk of developing delirium, cognitive and functional impairment as suggested by some authors during preoperative assessment in older-frail adults [11].

In conclusion, our case shows that the shared decision-making process related to patients with severe disability and significant frailty, who present high mortality rates even after any-stress surgery, requires a personalized and multidisciplinary approach (doctors, nurses, social and health workers, caregivers) evaluating the appropriateness of a surgical procedure and focuses on optimizing patient outcome.

### Follow-up

About 6 months after the first diagnosis of renal neoplasm, the patient shows no sign of physical or mental suffering; he can participate in the small events and parties organized by the facility of residence with satisfaction, and it was not necessary to activate any palliative care services.

## Data Availability

The datasets used are available from the corresponding author upon reasonable request.
